# Local Field Potential-Guided Contact Selection Using Chronically Implanted Sensing Devices for Deep Brain Stimulation in Parkinson’s Disease

**DOI:** 10.3390/brainsci12121726

**Published:** 2022-12-16

**Authors:** Joshua N. Strelow, Till A. Dembek, Juan C. Baldermann, Pablo Andrade, Hannah Jergas, Veerle Visser-Vandewalle, Michael T. Barbe

**Affiliations:** 1 Department of Neurology, Faculty of Medicine and University Hospital Cologne, University of Cologne, 50937 Cologne, Germany; 2 Department of Stereotactic and Functional Neurosurgery, Faculty of Medicine and University Hospital Cologne, University of Cologne, 50937 Cologne, Germany; 3 Department of Psychiatry and Psychotherapy, Faculty of Medicine and University Hospital Cologne, University of Cologne, 50937 Cologne, Germany

**Keywords:** Parkinson’s disease (PD), deep brain stimulation (DBS), nucleus subthalamicus (STN), local field potential (LFP), DBS programming

## Abstract

Intra- and perioperatively recorded local field potential (LFP) activity of the nucleus subthalamicus (STN) has been suggested to guide contact selection in patients undergoing deep brain stimulation (DBS) for Parkinson’s disease (PD). Despite the invention of sensing capacities in chronically implanted devices, a comprehensible algorithm that enables contact selection using such recordings is still lacking. We evaluated a fully automated algorithm that uses the weighted average of bipolar recordings to determine effective monopolar contacts based on elevated activity in the beta band. LFPs from 14 hemispheres in seven PD patients with newly implanted directional DBS leads of the STN were recorded. First, the algorithm determined the stimulation level with the highest beta activity. Based on the prior determined level, the directional contact with the highest beta activity was chosen in the second step. The mean clinical efficacy of the contacts chosen using the algorithm did not statistically differ from the mean clinical efficacy of standard contact selection as performed in clinical routine. All recording sites were projected into MNI standard space to investigate the feasibility of the algorithm with respect to the anatomical boundaries of the STN. We conclude that the proposed algorithm is a first step towards LFP-based contact selection in STN-DBS for PD using chronically implanted devices.

## 1. Introduction

The programming of stimulation settings remains a critical factor in the postoperative management of patients undergoing deep brain stimulation (DBS) for Parkinson’s disease (PD). However, currently, the determination of effective stimulation contacts and the optimization of stimulation parameters remains time and resource consuming [1,2]. Especially, the initial parameter setting is complex and requires highly specialized staff.

Despite compelling evidence that local field potential activity (LFP), or especially beta frequency activity, might serve as a valuable biomarker in PD [3,4,5,6,7,8,9,10,11,12,13,14,15,16], electrophysiologically guided contact selection has not been implemented into clinical routine yet. As most of the relevant studies mentioned above were limited to data that were extracted during intra- and perioperative lead externalization, electrophysiological examinations are not accessible to all clinicians and might be compromised due to lesion effects [17,18] and postoperative shifts or rotation of the DBS lead [19]. Additionally, due to the chronic progression of the disease, it is unclear whether the distribution and extent of beta activity remains stable over time. Thus, “bed-side” postoperative recordings obtained using chronically implanted devices might offer more reliable and clinically relevant signals to inform stimulation programming.

With the introduction of commercially available sensing devices, it is possible to record LFPs in addition to their therapeutic use as stimulators. To date, however, postoperative LFP recordings are restricted to bipolar contact configurations, which, without further anatomical information, impair the relating of recording results to monopolar stimulation configurations. Consequently, sensing capacities often remain unused in clinical practice.

This study aimed to develop a clinical algorithm that can be used to inform contact selection for STN-DBS in PD by clinicians unexperienced in the interpretation of electrophysiological data. We hypothesized that elevated beta activity can be used to determine clinically effective contacts. For this, we employed an automated approach that used the weighted average of bipolar recordings obtained using chronically implanted devices to determine effective monopolar contacts. In the first step, the algorithm identified the stimulation level with the highest beta activity. In the second step, based on the prior identified level, the directional contact with the highest activity was chosen. Contacts were rank-order-correlated to the clinical stimulation results as determined during standardized monopolar contact review. Additionally, to investigate the feasibility of the algorithm with respect to the anatomical boundaries of the STN, we considered the distance from the previously identified anatomical sweet spots of the STN after the reconstruction of the DBS leads. Finally, the results of contact selection with our newly established algorithm were compared to those of contact selection with the visual interpretation of bipolar signals and those of monopolar contact selection as performed in clinical routine. Our results suggest that this algorithm might serve as a useful tool to inform contact selection for DBS therapy in the future.

## 2. Materials and Methods

### 2.1. Patients

We included seven patients (14 hemispheres) from the University Hospital Cologne DBS center in this analysis (more details in supplementary Table S1). All patients underwent bilateral STN-DBS as per clinical routine and received a Medtronic Percept^TM^ PC neurostimulator with directional SenSight^TM^ leads, which are less prone to signal disturbances than previous electrode models (Medtronic, USA, Minneapolis, MN 55432). The study was carried out following the Declaration of Helsinki. All data were assessed as part of clinical routine. Due to the retrospective character of the study, informed patient consent was not necessary. This was approved by the institutional review board of University of Cologne (protocol number 22-1993).

### 2.2. Monopolar Review

As per routine in our center, the stimulation parameters were optimized during hospitalization three months (±six weeks) after surgery using monopolar contact review (MPR). After overnight withdrawal of medication (12 h for short-, 24 h for long-lasting dopaminergic medication), the clinical effect of each electrode contact (ring contacts, directional levels in ring mode and directional contacts; n = 10) was tested at a fixed amplitude of 2 mA, with a frequency of 130 Hz and a pulse width of 60 μs. Contacts were selected in a randomized order. The patient was blinded to the selected contact level and stimulation amplitude. The rater was unaware of the results of the electrophysiological examination. We assessed rigidity according to item 22 of Unified Parkinson’s Disease Rating Scale (UPDRS) Part III. The severity of akinesia was assessed according to the means of item 23 (finger tapping) and item 25 (hand rotation). Half-point steps increased the resolution of the rating. The clinical efficacy of each contact served as the primary outcome parameter and was calculated as the total difference between the sum of the baseline score (sum of all items assessed under the StimOFF-MedOFF condition) prior to testing and the sum of the assessed scores at the above-mentioned stimulation parameters for each electrode contact (StimON-MedOFF). All stimulation levels (n = 4) per hemisphere were then ranked from the lowest (rank #1) to the highest values (rank #4). Consecutively, directional contacts were ranked in a similar fashion, from the lowest (rank #1) to the highest (rank #3), for each directional level.

### 2.3. Electrophysiological Recordings and Signal Processing

Recordings were performed prior to clinical MPR. Raw LFP data were recorded at a sample rate of 250 Hz using the standardized Brainsense^TM^ Survey function of the Medtronic Percept^TM^ PC neurostimulator (Medtronic, USA, Minneapolis, MN 55432). For each hemisphere, all channels of the implanted lead (15 bipolar contact combinations per hemisphere) were recorded simultaneously for approximately 20 s. LFPs were recorded after a stimulation wash-out period of two minutes, while the patient being awake and at rest. Patients were instructed to sit comfortably without moving.

We used Welch’s power spectral density (PSD) estimate with a hamming window lasting 1 s, an overlap between segments of 50% and 100 frequency bins between 1 and 100 Hz with a resolution of 1 Hz/bin to calculate the PSD of each bipolar recording. Data were zero-padded, as the segment length fell below the 256-point fast Fourier transformation (FFT). The modified periodograms were averaged to obtain the PSD estimate. All data were analyzed using in-house MATLAB scripts (version 2022b; MathWorks, USA, Massachusetts, MA 01760).

All raw data (210 recordings) were visually controlled for contamination of movement artifacts and electrocardiographic (ECG) signal deflections (characteristic QRS-like signal deflections of approximately 150 ms). In 6 out of 7 patients, right-sided subclavicular IPGs were implanted (supplementary Table S1). No visual movement artifacts and no ECG contamination of the raw data were detected. This was confirmed after the usage of the openly available Perceive Toolbox with automatized ECG artifact detection (www.github.com/neuromodulation/perceive/, v0.2, 17 April 2021).

### 2.4. DETEC Algorithm

**Figure 1:**
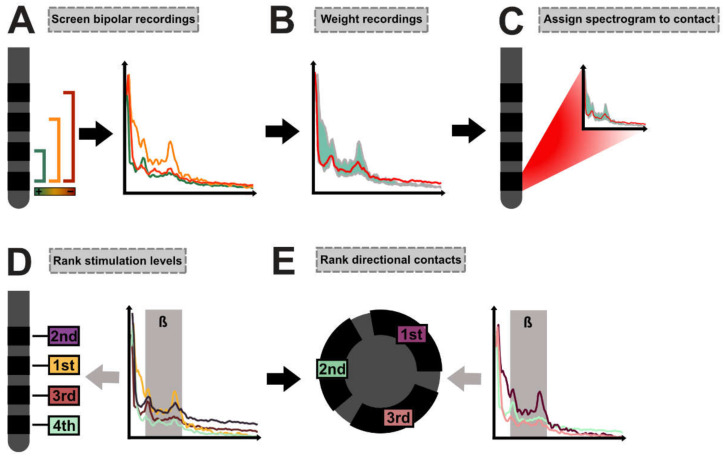
**DETEC algorithm:** (**A**) For each contact, all possible bipolar recordings from the respective contact were screened. (**B**) Bipolar recordings were weighted according to their distance from one another to obtain an average spectrogram of the respective monopolar contact. (**C**) The spectrogram was assigned to the according contact. (**D**) In the first step, beta activity (ß) across all electrode levels (n = 4) was ranked to identify the level with the highest activity. #4 = the lowest rank; #1 = the highest rank. (**E**) Similarly, in the second step, the directional contact with the highest beta activity was determined based on the prior identified stimulation level (n = 3). #3 = the lowest rank; #1 = the highest rank.

In the first step, for each contact, all bipolar recordings involving the respective contact were screened (Figure 1A). Bipolar recordings were then weighted by dividing the spectrograms with the absolute distance in millimeters between the center of the screened contact and its respective bipolar recording partner. The weighted average spectrogram was next calculated with the summation of the weighted spectrograms and division by the sum of the weights (Equation (1), Figure 1B).

(1) $$PSD_{weighted}=\frac{\sum_{i=1}^{n}PSD_{i}\;*\;\frac{1}{d_{i}}}{\sum_{i=1\;}^{n}\frac{1}{d_{i}}}$$

In Equation (1), *PSD_i_* is the *PSD* from bipolar recording *i* of the n bipolar recordings involving the investigated contact and *d_i_* is the distance between the center of the investigated contact and its bipolar recording partner for bipolar recording *i* (mm).

The weighted average spectrogram was then assigned to the screened contact (Figure 1C). We removed the aperiodic 1/f component as suggested by the FOOOF algorithm [20]. In the next step, we determined whether the averaged spectrogram of each contact depicted a peak within the beta band (13–35 Hz) or if the normalized activity within the low beta band(13–20 Hz) had to be used in further analyses. For this, we implemented an automated function that determined local maxima and evaluated whether the maxima met the following criteria: For each spectrogram, a non-linear least-squares curve fitting model was applied (best fit in means of least squares). If the activity of the identified local maximum exceeded the fitted data and its intrinsic height exceeded neighboring data points by at least the root-mean-square error (RMSE) of the curve fitting model, the maximum was identified as a peak (Supplementary Figure S1). If the algorithm detected a peak in at least one recording in a certain hemisphere (per patient and per hemisphere; n =10), normalized activity at this peak was used for the analysis of this hemisphere. If no peak was detected, the algorithm used normalized activity within the low beta range for the analyses (formulas are given in the supplementary materials). Depending on the respective analysis strategy (beta peak activity or activity within the low beta band), the stimulation levels were then ranked from the lowest (rank #1) to the highest values (rank #4) to determine the presumably best stimulation level (Figure 1D). Based on the previously identified stimulation level, the best directional contact on that level was chosen next. If no directional level was determined as the best stimulation level, the neighboring directional level was used for the analysis of the best directional contact. Again, based on the respective analysis strategy, contacts were ranked from the lowest (rank #1) to the highest values (rank #3) within the directional setting (Figure 1E).

### 2.5. Clinician-Evaluated Beta Power—Visual Approach

To determine how well the results of the automated algorithm would identify relevant beta activity compared with clinicians using the graphical user interface provided by the programming device, we also investigated a visual approach (“eyeballing”). The visual approach was performed by two experienced DBS clinicians at our center (M.T.B. and T.A.D.). The clinicians were given the following instructions to review bipolar recordings to detect relevant beta activity and assign it to a single monopolar contact:

Please determine the best stimulation level (ring level recordings only).

- Evaluate the beta peak between 13 and 35 Hz; if more than one peak is shown, please choose the one in the low beta frequency range (13–20 Hz);
- If no visible beta peak is present, please evaluate overall power in the low beta range;
- Compare beta peak or low beta power for each contact combination;
- Assign the highest activity to a single best level;
- Repeat for both hemispheres.

Based on the prior identified level, determine the best directional contact.

If no directional level was determined, choose the neighboring directional level.

- Evaluate the beta peak between 13 and 35 Hz; if more than one peak is shown, please choose the one in the low beta frequency range (13–20 Hz);
- If no visible beta peak is present, please evaluate power in the low beta range;
- Compare beta peak or low beta power for each contact combination;
- Assign the highest activity to a single directional contact (best directional).

Repeat for both hemispheres.

### 2.6. Statistical Analysis

The relationship between ranked activity in the beta frequency and ranked clinical efficacy of the contacts was assessed using non-parametric Spearman’s correlation. Ranked data were used to allow validation at the group level to be conducted. At the single-patient level, we fitted a least-squares line to depict the trend of the relationship (beta activity and clinical efficacy). However, this was purely illustrative and does not yield statistical relevance. To control whether our observation held true when using absolute values instead of ranks, we fitted a linear mixed-effects model using the absolute clinical outcome as the variable of interest, normalized activity within the beta frequency as the fixed effect and the individual hemisphere of patients as a random intercept effect. This was performed for all contacts investigated across the cohort (n = 140). To investigate whether the different approaches (monopolar review, DETEC algorithm and visual approach) would produce different clinical results, we performed the Kruskal–Wallis test. Here, the distribution of the mean clinical efficacy of the two best contacts (the best stimulation level and the best directional contact per patient and per hemisphere) as determined using the different approaches were chosen for analysis. We applied the Tukey–Kramer post hoc test that corrected for multiple comparisons and determined which groups significantly differed from the others if *p* was < 0.05. Samples were tested for normal distribution using the one-sample Kolmogorov–Smirnov test and visual interpretation of Q-Q plots. All statistical analyses were performed using MATLAB (version 2022b; MathWorks, Natick, MA, USA).

### 2.7. Imaging and Electrode Reconstruction

All patients received routine preoperative MRI and postoperative CT imaging (3T Philips Ingenia MRI system; Philips Medical Systems). The quality of preoperative MRI and postoperative CT images, as well as co-registration and normalization, was visually inspected by experienced DBS clinicians (T.A.D. and J.C.B.). DBS leads were localized using Lead-DBS software [21]. Briefly, preoperative MRI and postoperative CT images were first co-registered linearly and then non-linearly normalized into MNI ICBM 2009b NLIN ASYM template space using advanced normalization tools (ANTs). Co-registrations, as well as normalizations, were visually inspected for accuracy and refined if needed. An additional subcortical refinement step was added to correct for any brain shift caused by surgery. All DBS leads were pre-reconstructed using the PaCer method and manually refined if needed [22]. Finally, to determine the anatomically accurate positioning of the directional electrodes, we used the sequential DiODe-algorithm to correct the lead orientation [23]. Note that the DiODe-algorithm for directional Medtronic SenSight^TM^ leads has not been validated in a phantom study yet. The electrode reconstructions are given in supplementary Figure S2A.

### 2.8. Spatial Localization of Electrophysiological Recordings

To allow the pooling of electrophysiological data among patients to be conducted, activity values were z-scored according to their means and standard deviations. All activity values were then localized to the coordinates of the respective monopolar contact in MNI ICBM 2009b NLIN ASYM template space (geometric center for ring contacts/levels; center of the contacts surface for directional contacts). Electrode reconstruction was performed as described above. The coordinates of the directional contacts were shifted by 0.5 mm orthogonally to the DBS lead. This is in accordance with the notion that LFPs cover a spatial extent of several hundred microns around the electrode contact [24]. All coordinates and data points of the right hemisphere were non-linearly flipped onto the left hemisphere, and vice versa, and were finally pooled across hemispheres to enhance the number of data points for analysis [25,26]. This resulted in a point cloud corresponding to the distribution of beta activity values in anatomical space. For visualization purposes, a linear interpolant was calculated to estimate activity values in the empty space between coordinates. To validate the feasibility of the algorithm we calculated the distance between the coordinates and previously identified anatomical [27] and electrophysiological [26] sweet spots. The center of gravity (left hemisphere) as reported by the authors was used for calculation (anatomical sweet spot: x = −12.5, y = −12.72, z = −5.38; electrophysiological sweet spot: x = −12.2, y = −12.5, z = −6.4). We next fitted a linear mixed-effects model (LME) using beta activity values obtained using the DETEC algorithm as the variable of interest, the distance between the described sweet spots as the fixed effect and the sampling site (hemisphere per patient) as a random intercept effect.

## 3. Results

### 3.1. Determination of Effective Stimulation Level

**Figure 2:**
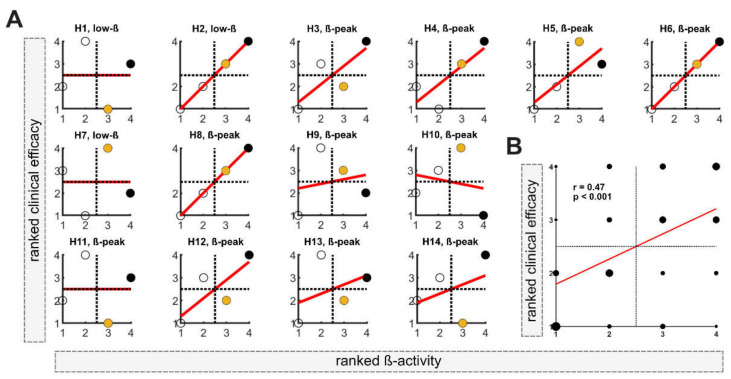
**Determination of the best stimulation level:** (**A**) Correlation between ranked clinical efficacy (improvement in UPDRS-III items 22, 23 and 25) and ranked ß-activity for each hemisphere (H1–H14). The filled black dots correspond to the level with the highest beta activity. The filled yellow dots correspond to the level with the second-highest beta activity. n per hemisphere = 4. #1 = the lowest rank; #4 = the highest rank. (**B**) At the group level, ranked beta activity correlated with ranked beta activity using Spearman’s correlation (n = 56). Larger dots indicate multiple data points. #1 = the lowest rank; #4 = the highest rank.

In the first step, the DETEC algorithm was used to identify the presumably best of the n = 4 stimulation levels in each of the n = 14 hemispheres based on ranked activity within the beta frequency (total n = 56). Ranked clinical efficacy and ranked beta activity are shown for each hemisphere (H1-H14) in Figure 2A. In 11 out of 14 hemispheres, a relevant beta-peak was detected and used for analysis. Contrarily, in 3 out of 14 hemispheres, normalized low beta activity was used (H1, H2 and H7). In all hemispheres, the stimulation level with either the highest or second-highest activity in the beta frequency band was located in the upper-right quadrant, matching the stimulation levels with good clinical efficacy. In 7 out of 14 hemispheres (50%), the stimulation level with the highest beta activity matched the level with the best clinical efficacy. When only considering hemispheres with a relevant beta peak, in 6 out of 11 hemispheres, the highest beta activity matches the most clinically efficient contact and in 2 out of 11 matches the second-best clinical level. At the group level, Spearman’s correlation among all evaluated levels indicated a moderate relationship between beta activity and clinical efficacy (r = 0.47; *p* < 0.001; Figure 2B).

### 3.2. Determination of Best Directional Contact

**Figure 3:**
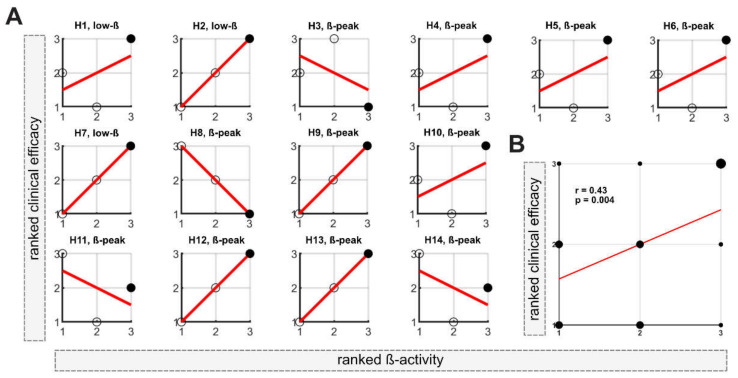
**Determination of the best directional contact:** (**A**) Correlation between ranked clinical efficacy (improvement in UPDRS-III items 22, 23 and 25) and ranked weighted ß-activity for each hemisphere (H1-H14). The filled black dots correspond to the level with the highest beta activity. n per hemisphere = 3. #1 = the lowest rank; #3 = the highest rank. (**B**) At the group level, ranked beta activity correlated with ranked beta activity using Spearman’s correlation (n = 42). Larger dots indicate multiple data points. #1 = the lowest rank; #3 = the highest rank.

In the second step, the DETEC algorithm was used to identify the presumably best directional contact based on the previously defined stimulation level with the highest beta activity. If no directional level was determined as having the highest beta activity, the neighboring directional level was used to identify effective directional contacts. Figure 3A depicts the relationship between the ranked clinical efficacy and ranked beta activity for each directional contact of the respective hemisphere (H1–H14). In 10 out of 14 hemispheres (71%), the directional contact with the highest beta activity matched the contact with the highest clinical efficacy. In 4 out of 14 hemispheres (29%), the algorithm failed to detect the most clinically effective contact for that level (Figure 3A). At the group level, Spearman’s correlation among the ranked data of all directional contacts evaluated using the algorithm (n = 42) indicated a moderate relationship between activity in the beta frequency range and clinical efficacy (r = 0.43; *p* = 0.004; Figure 3B).

### 3.3. Beta Activity as a Clinical Predictor of DBS Efficacy

**Figure 4:**
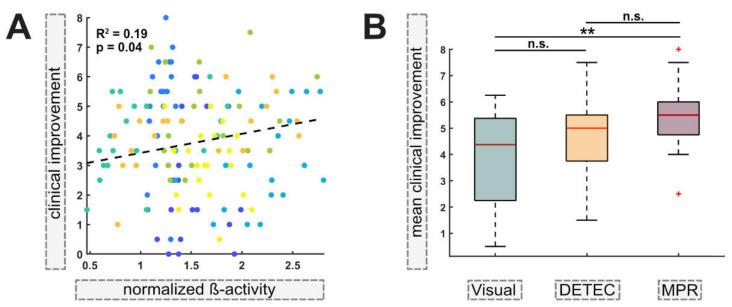
**Beta activity as a predictor for clinical efficacy:** (**A**) A linear mixed-effects model between absolute improvement in UPDRS-III items 22, 23 and 25, and ß-activity (normalized activity) for each contact (n = 140) yielded a statistically significant relationship when controlling for the individual intercept per hemisphere, explaining R^2^ = 19% of variance within the model. The colors of the dots correspond to one responding (individual) patient. The dashed line represents the least-squares line of the overall model. (**B**) The boxplots show the distribution of the mean clinical improvement (improvement in UPDRS-III items 22, 23 and 25) of the best stimulation level and the best directional contact (two contacts per patient per hemisphere; n = 28) as determined using the visual approach, the algorithm and monopolar contact review (MPR). The Kruskal–Wallis test indicated a statistically significant difference among groups (chi-square = 9.71; *p* = 0.008; df = 2). The Tukey–Kramer post hoc test corrected for multiple comparisons if p was < 0.05. The red lines indicate the medians. The red crosses indicate outliers. ** *p* < 0.01.

In our previous analysis, we differentiated between the use of stimulation levels and directional contacts, as this is imbedded in the DETEC algorithm. Further, only ranked data were used to evaluate the relationship between elevated beta activity and clinical efficacy. To test whether this relationship held true using absolute values across all contacts (n = 140), we performed a linear mixed-effects model that controlled for differences in the intercept of each patient as the random effect. Within the model, higher clinical improvement was statistically significantly associated with elevated beta activity, explaining R^2^ = 19% (*p* = 0.04; Figure 4A) of the variance within the model. This indicates that with increased activity, stimulation of the respective monopolar contacts yields higher clinical efficacy when controlling for inter-patient differences.

### 3.4. Comparing Different Approaches

Above, we present that the DETEC algorithm can be used to inform contact selection via elevated activity in the beta frequency indicating a linear relationship. To test how well our algorithm performed compared with experienced DBS clinicians in interpreting the results of the electrophysiological examination, we presented bipolar recordings as depicted on the clinician programmer (“visual approach”) to two experienced DBS clinicians (T.A.D. and M.T.B.) and compared these results to the results of contact selection via the DETEC algorithm and of the MPR. Using the non-parametric Kruskal–Wallis test to determine the statistical differences in the mean clinical effectiveness of the two best contacts (the best stimulation level and the best directional contact; two contacts per patient and hemisphere) as chosen using the algorithm (mean = 4.5; median = 5.0), by the two clinicians (mean = 3.9; median = 4.4) or with the MPR (mean = 5.3; median = 5.5), we identified a statistically significant difference among groups (chi-square = 9.71; *p* = 0.008; df = 2). The Tukey–Kramer post hoc test that corrects for multiple comparisons revealed that the distribution of the mean clinical improvement of contacts chosen using the MPR was significantly different from that of contacts chosen using the visual approach (*p* = 0.006) but not significantly differed from contacts chosen using the DETEC algorithm (*p* = 0.164). Further, no statistically significant differences between the visual approach and the DETEC algorithm were determined (*p* = 0.403; Figure 4B).

### 3.5. Spatial Localization of Beta Activity

**Figure 5:**
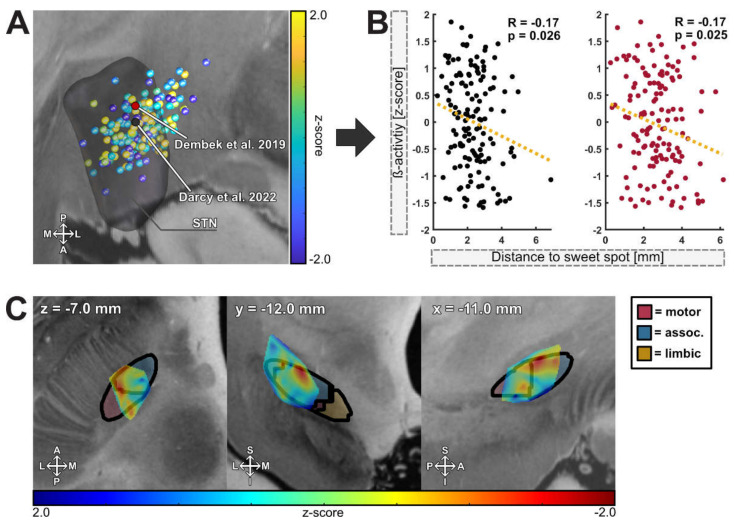
**Spatial localization of beta activity:** (**A**) Point cloud of beta activity distribution in common MNI space (left hemisphere). The coordinates are color-coded according to z-scored beta activity. The coordinate of the center of gravity of the anatomical sweet spot is depicted as a red dot (Dembek et al. 2019), and the center of gravity of the electrophysiological sweet spot is depicted as a black dot (Darcy et al. 2022). M = medial; L = lateral; S = superior, I = inferior. (**B**) Scattered activity values were correlated with their distance from the center of gravity of the anatomical (red) and electrophysiological sweet spots (black) of the left hemisphere (black; n = 140 contacts). (**C**) Peak values of beta activity in relation to subdivisions of the STN (Accolla et al. 2014). From left to right: axial (z), coronal (y) and sagittal (x) view. A = anterior; P = posterior; L = lateral; M = medial; S = superior, I = inferior.

The efficacy of DBS contacts has been correlated with proximity to anatomical boundaries and peaks of beta activity within the nucleus. To investigate the feasibility of our newly developed DETEC algorithm to measure and detect activity patterns around the DBS lead, we localized the activity values produced by the algorithm into common MNI space (Figure 5A) and calculated the distance from previously identified anatomical and electrophysiological sweet spots (Figure 5B). Here, elevated beta activity and distance from the previously described sweet spots was negatively correlated (anatomical sweet spot: R^2^ = 0.03, R = −0.17, *p* = 0.025; electrophysiological sweet spot: R^2^ = 0.03, R = −0.17, *p* = 0.025; Figure 5B), indicating that increased beta activity follows a distinct spatial distribution, which can be reliably reflected by the averaged spectrograms. Within the two models, R^2^ = 3% of the variance could be explained by the random intercept effect. Figure 5C depicts the interpolated data of this analysis. Here, the peak values of beta-mapping could be located within the dorsolateral portion of the STN at the MNI coordinates of x = −11.0 mm, y = −12.0 mm and y = −7 mm. When localized to an atlas that defines functional borders within the nucleus, peak beta activity was located at the dorsal border of the sensorimotor part, extending more anteriorly to the associative portion of the nucleus [28].

## 4. Discussion

In this study, we present a novel algorithm that uses bipolar recordings obtained with chronically implanted sensing devices to guide monopolar contact selection in STN-DBS for PD. Despite the well-established role of elevated activity within the beta frequency as a biomarker in PD, we here provide a comprehensible algorithm that might improve the accessibility of such information for clinicians unexperienced in the interpretation of electrophysiological data.

Overall, we could replicate recurring findings from the literature that used elevated activity within the beta band to guide postoperative contact selection [3,4,5,14,15,16] and showed that the distribution of peak beta activity followed a distinct anatomical pattern, similar to previously identified sweet spots [26,27]. As hypothesized, based on elevated beta activity, we could identify effective monopolar contacts. While the mean clinical efficacy of contacts chosen with the visual approach statistically differed, contact selection performed with the DETEC algorithm did not statistically differ from the mean clinical efficacy of contacts chosen in clinical routine. Given our results, we believe that this fully automated approach might offer a valuable, time-saving approach for clinical routine in the future.

### 4.1. Automated DETEC Algorithm

The influence of referencing remains a critical issue for studying the effects of EEGs and/or LFPs [24,29]. Prior studies solely comprised intraoperative, monopolar recordings (reference electrode within a distant tissue) in which, e.g., the stereotactic cannula could serve as a common reference. This allows the signal to be directly assigned to the responding contact. However, as already stated before, these recordings are only executed by a few DBS centers. In this study, we made use of a chronically implanted device that is, however, limited to bipolar montages between the stimulation contacts. As the produced signal is obtained as the difference between the electric potentials between both contacts, it is likely that the reference used is not neutral (due to its location within the STN), and meaningful components of the signal might be lost [30]. Bipolar recordings were shown to be helpful in an intraoperative setting during lead extension along the stereotactic trajectory; however, although currently the only alternative option, the interpretation of bipolar recordings in steadily implanted devices relies on careful visual inspection and requires immense electrophysiological expertise [5]. The issue of bipolar montages in DBS has been further discussed by Neumann and colleagues [31]. Additionally, a guide to electrophysiologically informed programming using commercially available sensing devices has been proposed by two renowned DBS centers [32,33]. However, our results suggest that the visual interpretation of bipolar montages produces high variance and might benefit from more objective control.

Consequently, in this study, we developed an algorithm that might overcome the issue of bipolar referencing and offer a fully automated approach to clinicians unexperienced in the interpretation of electrophysiological results. Within the algorithm, we employed all available bipolar montages between a single contact and its respective reference contact by calculating a weighted average signal as a function of the distance between them. With this, we penalize signals from contact combinations that are further apart and enhance the weight of local signals. This approach was inspired by Laplacian referencing for EEG [34], and our results suggest that this referencing scheme might be useful to develop averaged (monopolar) spectrograms. However, our algorithm has certain major disadvantages. Simplified, impedance is a measure of the size of the recording electrodes or contacts. Consequently, the geometry and impedances of the recording contacts immensely affect the recorded LFP signal. Although this more likely applies to single-unit activity than to the LFP signals measured here, we can assume differences between the recorded signals of the directional and ring contacts based on their varying impedances [21,22]. We partly overcame this problem by separating the detection of the effective stimulation level in the first step and the identification of an effective directional contact in the second step. However, based on our results, i.e., the insufficient performance in detecting the most clinically effective stimulation contacts in some patients, this might be an issue of varying impedances between the outer ring contacts and the two central contacts, which are divided into smaller segments. However, this also applies to the interpretation of bipolar montages, and our results suggest that our algorithm might offer a valuable bridge between the visual interpretation of bipolar montages and the lengthy monopolar review performed in clinical routine. Conclusively, in this cohort, our algorithm was capable of identifying effective stimulation contacts; however, we advocate that the choice of the best stimulation level might be prone to differences in the impedance across the most distal (ring) and directional (segmented) contacts.

### 4.2. Electrophysiological Biomarkers for PD

It has been intensely discussed whether solely elevated activity within the beta frequency is sufficient as a single biomarker for PD, as this simplification comes with loss of information. However, due to the limited memory capacity of currently available sensing devices, the sampling rate of the recordings remains low. Consequently, the analysis of frequencies above Nyquist (>125 Hz) was not accessible, although, e.g., activity within the gamma frequency range has been established as a pro-kinetic signal [35] and might be useful to predict effective stimulation contacts [36]. Here, we only assessed the predictive power of elevated beta activity on akinetic–rigid symptoms of PD, but we did not analyze its effect on tremor and other symptoms. Consequently, as beta band activity evolved as a biomarker for exclusively akinetic–rigid symptoms of PD, we excluded one tremor-dominant patient due to his/her deviating clinical profile [37,38,39,40,41,42].

Conclusively, our algorithm focused on elevated beta activity as a single biomarker in PD, or especially low beta frequency, which has been shown to be more sensitive to treated and/or untreated symptoms of PD than high beta activity [9,43,44,45]. However, recent insights have revealed that the spatial localization of low and high beta activity show a similar distribution and that both might serve as a valuable biomarker for contact selection in STN-DBS for PD [26].

### 4.3. Imaging-Based Analysis of the DETEC Algorithm

To investigate the feasibility of our algorithm with respect to the anatomical boundaries of the STN, we implemented an imaging-based analysis, including the localization of activity values and its relation to previously identified anatomical and electrophysiological sweet spots in common MNI space. Although our results indicated that beta activity distribution followed a distinct spatial pattern similar to previously identified sweet spots (Figure 5), this approach bared further limitations. The exact positioning of DBS leads without histology may be prone to distortion in MR imaging [46], susceptibility to the artifact of the DBS lead [47] and the accuracy of the normalization, co-registration and refinement processes [48]. However, the reconstruction via Lead-DBS software has been validated in manifold publications, including the latter on electrophysiological sweet spots [25,26].

### 4.4. Strengths and Limitations

A major limitation of this study was its small sample size. A post hoc power analysis was conducted using G*Power3 [49] to estimate the power achieved in this study. Using a parametric one-way ANOVA with fixed effects, a medium effect size of dz = 0.4 and an alpha of 0.5, the results revealed that this study achieved a power of 0.42. Given the small sample size in this study, results may be underpowered. However, the number of investigated contacts across the cohort was significantly higher, and previous studies showed reliable results with similar sample sizes. Further, one must note that the referencing scheme used here describes a novel approach that needs further validation. Future studies could benefit from technological advances that might allow a common referencing scheme to be obtained and consequently monopolar montages to be used. Additionally, not solely increased beta-activity alone, but also its suppression during stimulation ramping could be used as an additional tool for contact selection in the future [13]. Lastly, in this study, we used a modified version of the classic monopolar contact review, assessing clinical efficacy (described as absolute improvement from StimOFF-MedOFF to StimON-MedOFF in UPDRS-III items 22, 23 and 25) with a fixed amplitude of 2 mA rather than assessing the therapeutic window with regards to a single symptom (UPDRS-III item 22) in 0.5 mA increments as proposed by others [43,44]. However, we believe that this approach reflects the general motor improvement more precisely than investigation with regard to a single symptom.

In summary, our approach is a crucial step in implementing recordings obtained using chronically implanted sensing devices for clinical decision making by offering a comprehensible, fully automated algorithm. Although these results come from a relatively small sample size, they underline the clinical utility of postoperative electrophysiological examinations in identifying effective directional contacts in STN-DBS for PD. Despite the above-described disadvantages of postoperative, bipolar recordings, using such information may also benefit from overcoming possible signal derogation from intraoperative stun effects [18] and deviations from the intended implantation location and/or orientation in the postoperative period [19,23,50]. However, we advocate to validate our algorithm in independent, larger samples using a strongly confounder-controlled design.

## 5. Conclusions

This study provides a proof of principle that bipolar, postoperative, resting-state recordings obtained using chronically implanted sensing devices can be used to guide the initial parameter setting in STN-DBS for PD. By making our results publicly available, we advocate complementary studies using our or similar automated approaches. In this light, our results represent a first step towards improving the accessibility of such information for clinicians unexperienced in the interpretation of electrophysiological data.

## Data Availability

All in-house MATLAB scripts used for analysis are freely available within Open Science Framework (DOI:10.17605/OSF.IO/94YFW). Raw data are available upon reasonable request from the corresponding author.

## Supplementary Materials

The following supporting information can be downloaded at: https://www.mdpi.com/article/10.3390/brainsci12121726/s1, Figure S1: Automated peak detection; Table S1: Patient demographics and additional information; Figure S2: Electrode reconstructions of the cohort.
