# Supplementary material for: Local Field Potential-Guided Contact Selection Using Chronically Implanted Sensing Devices for Deep Brain Stimulation in Parkinson’s Disease
Source: Brain Sci. 2022 Dec 16;12(12):1726. doi: 10.3390/brainsci12121726 (PMC9776002; doi:10.3390/brainsci12121726)
Supplement: Supplementary file 1 [file brainsci-12-01726-s001.zip › brainsci-2088723-supplementary.pdf]

brain sciences

MDPI

Supplementary Material

Methods

Beta normalization

$$normalized\ beta - peak = \frac{mean\ power\ around\ the\ ß{-}peak\ (\pm 2Hz)}{mean\ power\ in\ the\ ß{-}band\ (13{-}15\ Hz)} \quad (1)$$

$$normalized\ low - beta = \frac{mean\ power\ in\ the\ low\ ß{-}band\ (\pm 2Hz)}{mean\ power\ in\ the\ ß{-}band\ (13{-}15\ Hz)} \quad (2)$$

Peak detection

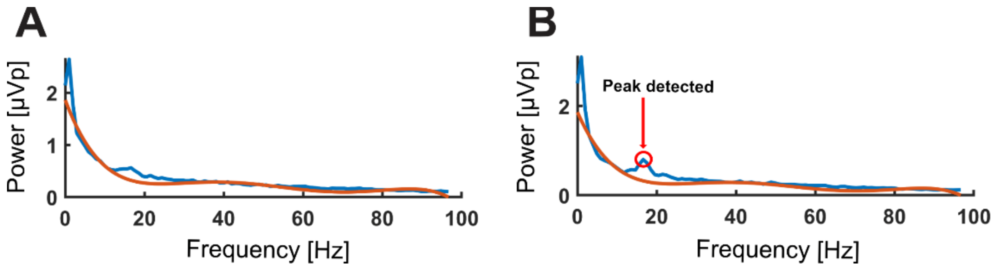

Supplementary figure S1: Automated peak detection. All weighted spectrograms from the respec-tive electrode were screened. Next, a non-linear least-squares curve fitting model is applied. If the power of the local maxima exceeds the fitted data and its intrinsic height exceeds neighboring data points for at least the root-mean-square error (RMSE) of the model, local maxima are characterized as peak. **(A)** No peak is detected by the algorithm. **(B)** The local maximum meets the peak criteria and is characterized as such. Blue = weighted spectrogram; orange = non-linear curve fitting model; red circle = peak detection.

Results

Patient demographics

Supplementary table S1: Patient demographics and additional information

	**Patient 1**	**Patient 2**	**Patient 3**	**Patient 4**	**Patient 5**	**Patient 6**	**Patient 7**	**Patient 8**
**Gender**	f	f	f	m	m	m	m	f
**Age (years)**	63	66	71	59	56	66	54	49
**Disease duration (years)**	9	15	8	17	16	17	7	8
**Disease laterality**	right	right	left	left	right	right	right	right
**Subtype**	EQ	EQ	AR	EQ	EQ	TD	EQ	EQ
**IPG placement**	right	right	left	right	right	left	right	right
**ß-peak (LH)**	No peak	14 Hz	17 Hz	No peak	19 Hz	No peak	25 Hz	26 Hz
**ß-peak (RH)**	No peak	18 Hz	16 Hz	15 Hz	18 Hz	No peak	25 Hz	25 Hz

1. AR, akinetic-rigid; TD, tremor dominant; EQ, equivalent; IPG, impulse generator. Patietn 6 was excluded from analysis.

A

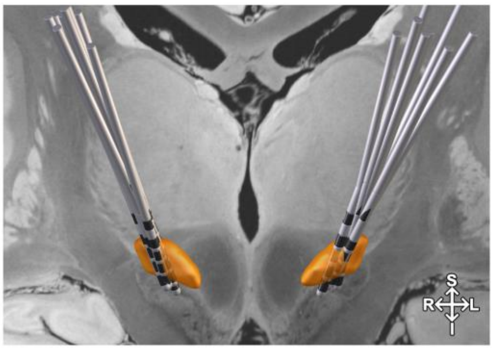

Supplementary figure S2. Comparison of different approaches. (A) Visualization of the cohort in MNI standard space (n = 8). Coronal view. Electrode models = Medtronic SenSight™ directional lead. DBS electrodes were localized using the advanced processing pipeline in Lead-DBS software using the TRAC/CORE approach and were manually refined if needed. Bilateral lead positions are projected on the STN (orange) as implemented in the DISTAL atlas (Ewert et al. 2018). Visualization is superimposed on a 7 Tesla MRI of the ex vivo human brain (Edlow et al. 2019).
